# Hyperglycaemia and its related risk factors in Ilam province, west of Iran- a population-based study

**DOI:** 10.1186/s40200-015-0203-9

**Published:** 2015-10-23

**Authors:** Khairollah Asadollahi, Ali Delpisheh, Parisa Asadollahi, Ghobad Abangah

**Affiliations:** Department of Social Medicine, Faculty of Medicine, Ilam University of Medical Sciences, Ilam, Iran; Psychosocial Injuries Researches Center, Ilam University of Medical Sciences, Ilam, Iran; Microbiology Researches Center, Ilam University of Medical Sciences, Ilam, Iran; Department of Gastroenterology, Faculty of Medicine, Ilam University of Medical Sciences, Ilam, Iran

**Keywords:** Hyperglycaemia, IFG, Diabetes, Ilam, Iran

## Abstract

**Background:**

Impaired fasting glycaemia (IFG) has been defined as the fasting plasma glucose level between 6.1 (110 mgl/dl) and 6.9 mmol/l (125 mgl/dl). Control of hyperglycaemia during acute illness among diabetic and non-diabetic patients has been associated with improved outcome. The aim of this study was to investigate the prevalence of and factors related to hyperglycaemia, IFG and diabetes in west of Iran.

**Methods:**

This project was performed by a cross-sectional method in Ilam province including 2158 people ≥ 25 years old. From the list of all rural and urban health centers of each county, several were randomly selected. For each selected health centre, families numbered 1–20 completed questionnaire forms for all the members aging ≥ 25 years. FBS was measured for all the participants by standard method. All the demographic and laboratory results were analysed using SPSS 16. Descriptive and regression analysis were used for statistical analysis appropriately.

**Results:**

A total of 2158 people were evaluated in this study, among which 72 % were female with a mean age of 45.5 ± 14 years. 40 % of participants were from urban regions and the mean height, weight, FBS and waist size of the participants were respectively as follows: 164 ± 8.9 cm, 68.4 ± 12.3 kg, 5.7 ± 2.8 mmol/l (102.6 ± 49.9 mg/dl) and 82.3 ± 14.3 cm. The prevalence of IFG, diabetes and hyperglycaemia among participants were 7.8 %, 11.8 % and 19.6 %, respectively and participants from urban area showed a significantly higher prevalence of hyperglycaemia compared to rural regions (*P* < 0.0001).

**Conclusion:**

The most effective factors associated with IFG and diabetes were family history of diabetes, age, hypertension, marital status, place of life and smoking, respectively. The prevalence of IFG, diabetes and hyperglycemia among the population living in Ilam province, west of Iran, were 7.8, 11.8 and 19.6 % respectively which were directly increased with age.

## Introduction

Plasma glucose is a continuous variable, with no definite “cut off” between normal and abnormal levels. High levels are usually associated with diabetes mellitus; however, relatively high levels may be detected in hospitalised patients in the absence of diabetes. Diagnostic criteria for diabetes are based on epidemiological evidences that identify levels, which predict the risk of future complications. Current diagnostic criteria were recently revised by the World Health Organisation (WHO), and include a plasma glucose level over 7.0 mmol/l (126 mgl/dl) (Fasting) or over 11.1 mmol/l (200 mgl/dl) (random). “Borderline” states of glucose intolerance are well recognised. Impaired glucose tolerance (IGT) [1] is diagnosed when the fasting plasma glucose is <7.0 mmol/l (126 mgl/dl), and 2-hour post-glucose load level is between 7.8 (140 mgl/dl) and 11.0 mmol/l (199 mgl/dl). More recently, “impaired fasting glycaemia” (IFG) has been defined, where the fasting plasma glucose level is between 6.1 (110 mgl/dl) and 6.9 mmol/l (125 mgl/dl) [1]. Both IGT and IFG probably represent states of glucose intolerance, which carry an increased risk of the future development of type 2 diabetes, as well as of cardiovascular diseases [2].

Some, but by no means all, patients admitted with hyperglycaemia have diabetes, so either known or unknown diabetes may be a cause of admission hyperglycaemia. There are evidences that at least some of these patients may have previously had undiagnosed diabetes [3].

Hyperglycaemia is common in hospital in-patients. A study from the USA has shown that more than one-third (38 %) of all patients admitted to an urban general hospital had fasting blood glucose levels exceeding 7.0 mmol/l, or 2 or more random blood glucose levels exceeding 11.1 mmol/l [4]. Another study investigated unrecognised diabetes among patients with coronary diseases and found that 46.6 % of the patients had hyperglycaemia with no prior history of diabetes [5].

In the UK, about 1.4 million people are known to have diabetes (about 3 % prevalence) and another million (2 % of the population) have undiagnosed diabetes [6]. This prevalence is grossly similar to that in other countries. Studies from developed countries reported prevalence rates of 7–38 % and mortality rates of 7.6–16 % associated with diabetes among general hospital admissions [4, 5, 7–12]. Globally, it was estimated that 382 million people suffered from diabetes with a prevalence rate of 8.3 % in 2013. North America and the Caribbean are the regions with the higher prevalence rates of diabetes with a figure of 11 %, followed by the Middle East and North Africa with a prevalence rate of 9.2 % and Western Pacific regions, with a prevalence rate of 8.6 %, closed to the global prevalence of diabetes. The number of people with diabetes is expected to rise to 592 million by 2035. Most people with diabetes live in low and middle-income countries which will experience the greatest increase in cases of diabetes over the next 22 years [13].

Although diabetes mellitus and impaired glucose tolerance are worldwide health problems, hyperglycaemia alone can also create major health disorders. These disorders involve people either in developed or developing countries. Studies from developing countries [14–16] broadly show similar prevalence rates of diabetes to those found in developed nations; however, this is changing. For example a study from urban Colombian community showed that glucose intolerance was common and noted that it was likely to increase in the future urbanization and aging of the population [12]. This is a major concern in all the developing nations.

Treatment of diabetes involves medicines, diet, and exercise to control blood glucose and prevent symptoms and problems. Keeping an ideal body weight and an active lifestyle may prevent type 2 diabetes. There is no way to prevent type 1 diabetes. The current population-based study was launched to investigate the prevalence of and factors related to hyperglycaemia, IFG and diabetes in Ilam province in the west of Iran.

## Methods

### Study population

This project was performed by a cross-sectional method in Ilam province, which included 2158 cases ≥ 25 years old during 2011–2012. Ilam province is located at the west part of Iran with about 450 km line border with Iraq and, according to the census of 2012, with a population of 557,599 people. Rural and urban population of this province are 215,040 and 342,559 and their relevant number of people ≥ 25 years old are 107,052 and 173,367 respectively.

### Sampling method

The sample size needed for this project was estimated for 2158 people. The samples were selected by a multi-stage sampling method in which a population quota was firstly allocated to the rural and urban levels in Ilam province, considering the last national census in 2007. At the next step, the list of all rural and urban health centers of Ilam province was taken from Ilam University of Medical Sciences. There are 63 active health centers, including 31 rural and 32 urban, in Ilam province and according to the number of rural and urban health centers of each county and their related population, several health centers were selected randomly (simple randomization) as block levels in both urban and rural areas. Totally, 19 health centers including 9 from rural and 10 from urban areas were selected for this study that was about one third of all active health centers in the province. Then for each selected health centre, families’ health dossier numbers 1–20 were chosen as household levels and investigators completed the questionnaire forms for all the selected family members aging ≥ 25 years old. If any selected family was absent for completing the questionnaire or did not agree to participate in the study, the first house after the family dossier number of 20 was substituted. An adjustment for design effect and correlations among clusters was applied in this study. Participants were informed about the study objectives and were entered into the study if they accepted their participation orally.

### Data collection procedure

All the participants who agreed to participate in the study were requested to attend in the nearest health centre for interview and laboratory test and those who could not attend or did not consent, were excluded. The information related to fasting blood sugar (FBS) taken and readiness to this test (8–12 h fasting time before taking a blood sample) had already been prepared in a sheet and was submitted to each family at the day before the test. The importance of fasting before the test was explained, face to face, by questioners during the distribution of information sheets. If, for any reasons, the fasting was broken by the participants, the candidate person was excluded from the study.

Each participant was taken 2–3 cc vein blood sample after which the specimens were centrifuged and separated within 30–45 min following collection. The plasma of each sample was separated by centrifugation at 1542 × g (~3000 rpm)/5 min (room temperature) with an interval time of plasma separation of 2 h. The separated samples were then transferred to Ebnsina laboratory by cold packs or dry ice within 30 min to maximum 3 h, depending on the distances between different counties and the laboratory centre. The FBS results equal to 7 ± 0.17 mmol/l (126 ± 3 mg/dl) were retested for certainty and a FBS ≥7 mmol/l (126 mg/dl) was considered as abnormal. Quality control was assayed each time the glucose method was performed by Westgard rules [17]. According to these rules controls were analyzed at the beginning of each run, periodically throughout and at the end of the run.

Demographic data were collected via interview and completing a validated questionnaire including age, sex, place of life, marital status, history of hypertension, family diabetes, daily exercise, smoking, weekly consumption of fruit, or animal oil. The data related to weight was then collected using a calibrated electronic digital scale, accurate to 100 g (Soehnle, Murrhardt, Germany) with subjects wearing only light underwear. Heights were measured to the nearest 0.1 cm using a Seca portable stadiometer while wearing no shoes and waist size was measured using a cloth tape measure by placing the tape measure midway between the hip bone and the bottom of lower ribs under the dresses.

Respondents were asked to report the frequency of consumption of a given serving of fruits or animal oil on a daily, weekly and monthly basis and data were then converted to weekly intake frequency and for daily exercise, participants were asked to report their daily or weekly physical activities (as hours) regardless of activities associated with their jobs and then converted to daily exercise.

### Study definitions

Hyperglycemia was considered as an abnormally (greater than the upper limit of the normal range) high concentrations of glucose in the circulating blood. In our study, hyperglycemia comprised IFG (pre-diabetes), unknown and confirmed diabetes.

World Health Organization (WHO) criteria for impaired fasting glucose differs from the American Diabetes Association (ADA) or Diabetes UK criteria, because the normal range of glucose is defined differently by each. Fasting plasma glucose levels of 5.5 mmol/l (100 mg/dl) and higher have been shown to significantly increase the complication rates; however, WHO opted to keep its normal upper limit under 6.1 mmol/l (110 mg/dl) to avoid the diagnosis of too many people with impaired fasting glucose, whereas the ADA and Diabetes UK lowered the normal upper limit to a fasting plasma glucose under 100 mg/dl [18–20].

- The normal range by WHO criteria: fasting plasma glucose level lower than 6.1 mmol/l (110 mg/dL)
- The normal range by ADA criteria: fasting plasma glucose level lower than 5.6 mmol/L (100 mg/dL)
- The normal range by Diabetes UK criteria: fasting plasma glucose level from 3.9 mmol/l (70 mg/dL) to 5.5 mmol/L (100 mg/dL)

### Data analysis

All demographic and laboratory results were entered into a validated form and were then analysed using SPSS 16. Descriptive and regression analysis were used to estimate the prevalence of IFG, diabetes, hyperglycaemia and the effects /weights of each variable on these abnormalities. Descriptive tables were used to show the results and the mean value of different variables were compared using *t*-test or Chi squared accordingly.

### Ethical considerations

This study was approved by ethic committee of Ilam University of Medical Sciences and was also financially supported by this university. Patients were entered into the study freely and did not pay for their FBS tests.

## Results

A total of 2158 people were evaluated in this study, among which 72 % were female with a mean age of 45.5 ± 14 years. 40 % of the participants were from rural regions and 60 % from urban area. The mean height (cm), weight (kg), FBS (mmol/l (mg/dl)) and waist (cm) of participants were as follows respectively: 164 ± 8.9, 68.4 ± 12.3, 5.7 ± 2.8 mmol/l (102.6 ± 49.9) and 82.3 ± 14.3. Most participants were married with a figure of 91.5 %. The prevalence of IFG, diabetes and hyperglycaemia among participants were 7.8 %, 11.8 % and 19.6 %, respectively and participants from urban area showed a significantly higher prevalence of hyperglycaemia than rural regions (*P* < 0.0001) (Table 1). The prevalence rates of IFG, diabetes and hyperglycemia, all showed a positive relationship with age among the participants (Table 2). The comparisons between different levels of plasma glucose and the participants’ weight or waist size are available in Tables 3 and 4.

**Table 1:** Frequency of different variables related to the participants of the diabetes study in Ilam Province

Variable		Mean ± SD	Number (%)	Outcome	
				Yes	No
Age (years)		45.5 ± 14	-	-	-
Height (Centimeter)		163.9 ± 8.9	-	-	-
Weight (kg)		68.4 ± 12.3	-	-	-
FBS mmol/l (mg/dl)		5.7 ± 2.8 (102.6 ± 49.9)	-	-	-
Waist size (Centimeter)		82.3 ± 14.3	-	-	-
Gender	Male	-	602 (28)	-	-
	Female	-	1553 (72)	-	-
Place of life	Urban	-	1288 (60)	-	-
	Rural	-	870 (40)	-	-
Marital status	Unmarried	-	98 (4.5)	-	-
	married	-	1974 (91.5)	-	-
	Widow	-	60 (3)	-	-
History of HT		-	-	238 (11 %)	1912 (89 %)
Weekly serving of fruits		-	-	2082 (97 %)	72 (3 %)
History of family diabetes		-	-	192 (9 %)	1901 (88 %)
Daily exercise		-	-	1822 (85 %)	322 (15 %)
Smoking		-	-	103 (5 %)	2052 (95 %)
Weekly serving of animal oil		-	-	567 (26 %)	1581 (74 %)

**Table 2:** Frequency of participants according to the different plasma glucose levels and related prevalence

Variable		Number of patients according to plasma glucose level (mg/dl)^a^					IFG ^b^Pr% (95%CI)	Diabetes Pr% (95%CI)	Hyper-glycaemia Pr% (95%CI)	P value
		<70	70–110	111–125	≥126	Total				
Place of life	Rural	36	707	46	81	870	5.3 (4.7–5.6)	9.3 (8.9–9.6)	14.6 (14.3–15.0)	0.000
	Urban	86	907	122	173	1288	9.5 (9.1–10.0)	13.4 (12.8–13.7)	22.9 (22.0–23.6)	
	Total	122	1614	168	254	2158	8.2 (7.8–8.6)	11.8 (11.3–12.2)	20 (19.2–20.9)	
Gender	Male	27	447	55	73	602	9.1 (8.6–9.5)	12.1 (11.7–12.6)	21.2 (20.6–21.9)	0.324
	Female	95	1164	113	181	1553	7.3 (6.7–7.8)	11.7 (11.1–12.3)	19 (18.5–19.7)	
	Total	122	1611	168	254	2155	7.8 (7.2–8.3)	11.8 (11.4–12.1)	19.6 (19.1–20.2)	
Marital status	Married	113	1469	155	237	1974	7.9 (7.3–8.5)	12.3 (11.7–12.9)	20.2 (19.4–21.0)	0.033
	Unmarried	7	81	6	4	98	6.1 (5.5–6.7)	4.1 (3.5–4.7)	10.2 (9.8–10.7)	
	Widow	1	42	6	11	60	10 (9.3–10.7)	18.3 (17.8–18.7)	28.3 (27.1–29.2)	
	Total	121	1592	167	252	2132	7.8 (7.2–8.5)	11.8 (11.3–12.4)	19.6 (19.1–20.3)	
History of HT	Yes	5	157	25	51	238	10.5 (10.2–10.9)	21.4 (21.0–21.8)	31.9 (30.8–32.5)	0.001
	No	116	1450	143	203	1912	7.5 (7.1–7.9)	10.6 (10.3–11.0)	18.1 (17.7–18.5)	
	Total	121	1607	168	254	2150	7.8 (7.4–8.2)	11.8 (11.4–12.3)	19.6 (19.1–20.1)	
Weekly using of fruit	Yes	114	1556	164	248	2082	7.9 (7.4–8.3)	11.9 (11.4–12.6)	19.8 (19.1–20.6)	0.000
	No	8	54	4	6	72	5.6 (5.1–5.9)	8.3 (7.8–8.8)	13.9 (13.0–14.7)	
	Total	122	1610	168	254	2154	7.8 (7.4–8.3)	11.8 (11.1–12.7)	19.6 (18.9–20.3)	
History of family diabetes	Yes	14	107	15	56	192	7.8 (7.5–8.1)	29.2 (28.3–29.6)	37 (36.5–37.6)	0.000
	No	108	1456	50	187	1901	2.6 (2.3–3.0)	9.8 (9.4–10.2)	12.4 (11.9–12.8)	
	Total	122	1563	165	243	2093	7.9 (7.5–8.3)	11.6 (11.1–12.0)	19.5 (18.9–20.1)	
Daily exercise	Yes	104	1382	134	202	1822	7.4 (7.0–7.9)	11.1 (10.6–11.5)	18.5 (18.1–19.2)	0.000
	No	18	219	34	51	322	10.6 (10.3–11.0)	15.8 (15.1–16.7)	26.4 (25.2–27.4)	
	Total	122	1601	168	253	2144	7.8 (7.4–8.1)	11.8 (11.0–12.7)	19.6 (18.8–20.4)	
Smoking	Yes	4	73	12	14	103	11.7 (11.3–12.2)	13.6 (13.1–14.0)	25.3 (24.6–26.1)	0.000
	No	118	1538	158	240	2052	7.7 (7.2–8.1)	11.7 (11.2–12.1)	19.4 (18.6–20.2)	
	Total	122	1611	168	254	2155	7.8 (7.5–8.3)	11.8 (11.1–12.4)	19.6 (18.7–20.5)	
Weekly using of animal oil	Yes	24	428	53	62	567	9.3 (8.1–10.4)	10.9 (10.2–11.7)	20.2 (19.5–21.0)	0.028
	No	97	1177	115	192	1581	7.3 (6.2–8.1)	12.1 (11.5–12.8)	19.4 (18.6–20.5)	
	Total	121	1605	168	254	2148	7.8 (6.9–9.0)	11.8 (11.1–12.6)	19.6 (18.8–20.6)	
Age group (years)	25–40	69	724	52	59	904	5.8 (4.5–6.8)	6.5 (5.9–7.0)	12.3 (11.7–12.9)	0.000
	41–60	43	672	77	141	933	8.3 (7.1–9.5)	15.1 (14.4–15.7)	23.4 (22.1–24.9)	
	>60	10	218	39	54	321	12.2 (11.6–12.9)	16.8 (16.2–17.4)	29 (27.9–31.1)	
	Total	122	1614	168	254	2158	7.8 (6.7–9.6)	11.8 (11.2–12.8)	19.6 (18.1–20.9)	

*HT* hypertension, *FBS* fasting blood glucose

^a^FBS: <3.9 mmol/l = <70 mg/dl; FBS: 3.9–6.1 mmol/l = 70–110 mg/dl; FBS: 6.12–6.9 mmol/l = 90–125 mg/dl; FBS: ≥7 mmol/l (≥126 mg/dl)

^b^Pr: prevalence

**Table 3:** Plasma glucose level of participants of the Ilam diabetes study, according to their weight groups

Variables		Weight group (kg)							
		Under 50	50–60	61–70	71–80	81–90	91–100	≥100	Total
Plasma glucose level	<3.9 mmol/l (<70 mg/dl) (n)	9	27	46	46	10	4	0	122
	3.9–6.1 mmol/l (70–110 mg/dl) (n)	95	331	552	552	148	41	22	1568
	6.1–6.9 mmol/l (111–125 mg/dl) (n)	0	38	63	63	11	8	2	162
	≥7 mmol/l (≥126 mg/dl)	15	56	79	79	27	8	1	246
	Total (n)	119	452	740	740	196	61	25	2099
IFG prevalence% (95%CI)		0	8.4 (8.1–8.8)	8.5 (8.1–8.9)	8.5 (8.1–8.9)	5.6 (5.4–5.8)	13.1 (12.7–13.5)	8 (7.5–8.4)	7.7 (7.2–8.1)
Diabetes prevalence% (95%CI)		12.6 (12.2–12.9)	12.4 (12.0–12.8)	10.7 (10.1–11.2)	10.7 (10.1–11.2)	13.8 (13.4–14.3)	13.1 (12.6–13.5)	4 (3.3–4.6)	11.7 (11.2–12.1)
Hyperglycaemia prevalence% (95%CI)		12.6 (12.1–12.9)	20.8 (20.4–21.3)	19.2 (18.7–19.6)	19.2 (18.7–19.6)	19.4 (19.0–19.8)	26.2 (25.7–26.6)	12 (11.6–12.5)	19.4 (18.9–19.7)

**Table 4:** Plasma glucose level of participants of the Ilam diabetes study, according to their waist size groups and gender

		Men waist size group (cm)			
Variable		Under 94	94–102	>102	Total
Plasma glucose level	<3.9 mmol/l (<70 mg/dl) (n)	17	3	3	23
	3.9–6.1 mmol/l (70–110 mg/dl) (n)	224	62	37	323
	6.1–6.9 mmol/l (111–125 mg/dl) (n)	33	2	1	36
	≥7 mmol/l (≥126 mg/dl)	36	1	7	48
	Total (n)	310	68	48	426
IFG Prevalence% (95%CI)		10.65 (10.3–11.0)	2.9 (2.3–3.4)	2.1 (1.8–2.5)	8.5 (8.1–8.8)
Diabetes prevalence% (95%CI)		11.6 (11.3–12.1)	1.5 (1.1–2.0)	14.6 (14.2–14.9)	11.3 (10.9–11.7)
Hyperglycaemia prevalence% (95%CI)		22.3 (21.8–22.6)	4.4 (4.1–4.8)	16.7 (16.2–17.1)	19.7 (19.3–20.2)
Women waist size group (cm)					
Plasma glucose level	<3.9 mmol/l (<70 mg/dl) (n)	under 80	80–88	>88	Total
		49	17	17	83
	3.9–6.1 mmol/l (70–110 mg/dl) (n)	390	209	230	829
	6.1–6.9 mmol/l (111–125 mg/dl) (n)	42	20	15	77
	≥7 mmol/l (≥126 mg/dl)	57	37	28	122
	Total (n)	538	283	290	1111
IFG prevalence% (95%CI)		7.8 (7.1–8.2)	7.1 (6.7–7.5)	5.2 (4.9–5.6)	6.9 (6.4–7.3)
Diabetes prevalence% (95%CI)		10.6 (10.2–10.9)	13.1 (12.9–13.5)	9.7 (9.4–10.1)	11.0 (10.7–11.2)
Hyperglycaemia prevalence% (95%CI)		18.4 (18.0–18.7)	20.1 (19.7–20.6)	14.8 (14.3–15.5)	17.9 (17.5–18.3)

Logistic regression was applied for 12 different variables investigated in this study and 6 variables of family history of diabetes, age, hypertension, marital status, place of life and smoking were identified more frequently in relation with disturbances of serum glucose levels. The method used for logistic regression analysis was “enter” or standard regression analysis and all the categorical predictor values were firstly defined and selected in the model. The logistic regression was applied for diabetes, IFG and hyperglycemia, as dependent variables, in three separated times (each time one dependent variable) with the same method. The results of univariate and multivariate analysis are summarized in Tables 5 and 6. Four of the identified risk factors were evaluated by yes/no answers and the two variables of place of life and age were respectively evaluated based on whether the participants were living in a rural or urban area and if they were in an age group lower or higher than 50 years old. According to this analysis, the highest coefficient was related to positive history of diabetes, i.e., the possibility of having a serum glucose disturbance among those with a positive history of diabetes was more than those without. The availability rate of different variables to be included in the final analysis is shown by Table 7.

**Table 5:** Weighting given to different risk factors associated with diabetes, IFG and hyperglycaemia after univariate analysis

Type of disorder	Variable		Weighting	Exp (B), (95 % CI)	P value
Diabetes	Positive Family of diabetes	Yes	56.647	3.265, 2.87–3.66	0.000
		No^b^			
	Age	>50 years	35.126	2.224,1.71–2.89	0.000
		≤50 years^b^			
	History of hypertension	Yes	22.676	1.436, 1.14–1.83	0.000
		No^b^			
	Place of life	Urban	8.406	1.511, 1.14–1.99	0.004
		Rural^b^			
	At least 30 min daily exercise	Yes^b^	5.870	1.509, 1.08–2.11	0.015
		No			
	Married	Yes	5.615	2.731, 1.19–6.27	0.018
		No^b^			
	Female Waist size	>88 cm	3.169	0.779, 0.62–1.03	0.075
		≤88^b^cm			
	Male Waist size	>102 cm	2.321	1.332, 0.76–1.84	0.091
		≤102^b^cm			
	Weekly using of fruit	Yes^b^	0.846	0.672, 0.29–1.57	0.358
		No			
	Weekly using of animal oil	Yes	0.585	1.126, 0.83–1.53	0.444
		No^b^			
	Smoking	Yes	0.338	0.842, 0.47–1.5	0.561
		No^b^			
	Sex	Male	0.093	0.956, 0.72–1.28	0.761
		Female^b^			
	BMI	>25	0.063	1.034, 0.79–1.34	0.801
		≤25^b^			
IFG	History of hypertension	Yes	19.448	1.538,0.58–1.13	0.000
		No^b^			
	Place of life	Urban	18.685	1.672, 1.32–2.11	0.000
		Rural^b^			
	Male Waist size	>102 cm	12.331	1.812, 1.23–2.14	0.000
		≤102^b^cm			
	Female Waist size	>88 cm	9.881	1.691, 1.43–1.97	0.002
		≤88^b^cm			
	Married	Yes	8.728	2.687, 1.39–5.27	0.003
		No^b^			
	Age	>50 years	6.234	1.332, 1.06–1.67	0.013
		≤50 years^b^			
	Smoking	Yes	3.303	0.654, 0.41–1.03	0.069
		No^b^			
	Positive Family of diabetes	Yes	2.921	1.099, 0.74–1.63	0.089
		No^b^			
	Weekly using of animal oil	Yes	2.884	0.812, 0.64–1.03	0.638
		No^b^			
	At least 30 min daily exercise	Yes^b^	2.679	1.275, 0.95–1.71	0.102
		No			
	Sex	Male	2.135	0.838, 0.66–1.06	0.144
		Female^b^			
	Weekly using of fruit	Yes^b^	0.504	0.790, 0.41–1.52	0.478
		No			
	BMI	>25	0.286	1.061, 0.85–1.32	0.593
		≤25^b^			
Hyperglycaemia	Positive Family of diabetes	Yes	25.743	2.459, 1.93–2.86	0.000
		No^b^			
	Age	>50 years	39.798	1.846, 1.53–2.23	0.000
		≤50 years^b^			
	Place of life	Urban	32.358	1.758, 1.45–2.14	0.000
		Rural^b^			
	History of hypertension	Yes	19.448	1.538, 1.31–1.78	0.000
		No^b^			
	Married	Yes^b^	16.999	2.071, 1.8–5.24	0.000
		No			
	Male Waist size	>102 cm	16.684	1.731, 1.12–2.34	0.000
		≤102^b^cm			
	Female Waist size	>88 cm	15.255	1.681, 1.56–1.83	0.001
		≤88^b^cm			
	At least 30 min daily exercise	Yes^b^	9.508	1.475, 1.15–1.89	0.002
		No			
	Smoking	Yes	3.773	0.668, 0.44–1.04	0.052
		No^b^			
	Sex	Male	2.098	0.861, 0.71–1.65	0.148
		Female^b^			
	Weekly using of fruit	Yes^b^	1.550	0.704, 0.41–1.22	0.213
		No			
	BMI	>25	0.394	1.061, 0.88–1.26	0.530
		≤25^b^			
	Weekly using of animal oil	Yes	0.805	0.910, 0.74–1.12	0.370
		No^b^			

Exp (B) ^a^: Indicates the odds of having the disease (e.g., diabetes) among those with the risk factor (e.g., Family diabetes) compared to those without that risk factor. ^b^reference group

**Table 6:** Weighting given to different risk factors associated with diabetes, IFG and hyperglycaemia after multivariate analysis

Type of disorder	Variable		Weighting	Exp (B)^a^, (95 % CI)	P value
Diabetes	Positive Family of diabetes	Yes	48.862	3.616, 2.52–5.19	0.000
		No^b^			
	Age	>50 years	17.567	1.526, 1.22–1.84	0.000
		≤50 years^b^			
	History of hypertension	Yes	7.244	1.697, 1.15–2.53	0.007
		No^b^			
	Place of life	Urban	8.387	1.629, 1.37–1.89	0.004
		Rural^b^			
	Married	Yes	3.791	1.558, 1.24–2.31	0.041
		No^b^			
	Smoking	Yes	2.234	1.042, 0.099–1.44	0.048
		No^b^			
	Female Waist size	>88 cm	2.134	1.283, 0.92–1.83	0.144
		≤88^b^cm			
	Male Waist size	>102 cm	2.107	1.461, 0.87–2.44	0.147
		≤102^b^cm			
	Weekly using of fruit	Yes^b^	0.555	1.395, 0.58–3.35	0.456
		No			
	Weekly using of animal oil	Yes	0.542	0.886, 0.64–1.22	0.461
		No^b^			
	BMI	>25	0.324	0.9190.69–1.23	0.569
		≤25^b^			
	At least 30 min daily exercise	Yes^b^	0.318	0.899, 0.62–1.30	0.573
		No			
	Sex	Male	0.014	0.981, 0.71–1.35	0.905
		Female^b^			
IFG	Place of life	Urban	14.057	1.616, 1.48–1.79	0.000
		Rural^b^			
	Married	Yes	9.065	1.300, 1.137–1.66	0.003
		No^b^			
	Male Waist size	>102 cm	6.12	1.711, 1.12–2.62	0.013
		≤102^b^cm			
	Positive Family of diabetes	Yes	4.228	1.907, 0.61–1.35	0.03
		No^b^			
	Weekly using of animal oil	Yes	3.000	1.246, 0.97–1.68	0.083
		No^b^			
	Smoking	Yes	2.795	1.211, 0.74–1.97	0.095
		No^b^			
	Female Waist size	>88 cm	2.542	1.256, 0.96–1.65	0.098
		≤88^b^cm			
	Age	>50 years	2.272	0.824, 0.64–1.06	0.132
		≤50 years^b^			
	BMI	>25	2.157	0.838, 0.66–1.061	0.142
		≤25^b^			
	Sex	Male	2.015	0.831, 0.64–1.07	0.156
		Female^b^			
	Weekly using of fruit	Yes^b^	0.184	1.157, 0.65–2.25	0.668
		No			
	History of hypertension	Yes	0.718	1.171, 0.81–1.75	0.397
		No^b^			
	At least 30 min daily exercise	Yes^b^	0.273	0.919, 0.67–1.26	0.601
		No			
Hyperglycaemia	Positive Family of diabetes	Yes	27.876	2.154, 1.63–2.95	0.000
		No^b^			
	Age	>50 years	22.211	1.626, 1.504–1.78	0.000
		≤50 years^b^			
	History of hypertension	Yes	18.012	1.563, 1.15–2.13	0.005
		No^b^			
	Married	Yes	12.240	1.343, 1.19–1.63	0.000
		No^b^			
	Place of life	Urban	11.438	1.161, 1.45–1.77	0.000
		Rural^b^			
	Male Waist size	>102 cm	9.721	1.76, 1.23–2.51	0.002
		≤102^b^cm			
	Female Waist size	>88 cm	6.057	1.341, 1.06–1.75	0.014
		≤88^b^cm			
	BMI	>25	3.084	0.832, 0.68–1.02	0.079
		≤25^b^			
	Smoking	Yes	1.372	1.149, 0.74–1.79	0.542
		No^b^			
	Weekly using of animal oil	Yes	1.003	1.119, 0.89–1.44	0.317
		No^b^			
	Sex	Male	1.553	0.867, 0.69–1.15	0.213
		Female^b^			
	Weekly using of fruit	Yes^b^	0.770	1.293, 0.73–2.33	0.380
		No			
	At least 30 min daily exercise	Yes^b^	0.715	0.888, 0.68–1.17	0.398
		No			

Exp (B) ^a^: Indicates the odds of having the disease (e.g., diabetes) among those with the risk factor (e.g., Family diabetes) compared to those without that risk factor. ^b^: reference group

**Table 7:** The availability rate of different variables. ‘Classes’ indicates the percentage of participants falling within each variable group. Some classes do not sum to 100 % within each indicator because of missing data, so ‘Available results’ reports the percentage of patients for which data on that variable indicator was available

Variables		Number			Non-participation rate%
		Total	Classes%	Available results %	
FBS		2158	100	100	0
Weight		2101	97.4	97.4	2.6
Height		2104	97.5	97.5	2.5
Waist size	male	436	28.4	71.2	28.8
	female	1100	71.6		
Gender	Male	652	28	100	0
	female	1506	72		
BMI	<25	998	48	96.3	3.7
	25–30	870	41.9		
	>30	210	10.1		
Age group	25–40	898	41.8	99.5	0.5
	41–60	929	43.3		
	>60	321	14.9		
At least 30 min daily exercise	Yes	1822	85	97.4	2.6
	No	322	15		
History of hypertension	Yes	238	11.1	97.6	2.4
	No	1912	88.9		
Weekly using of animal oil	Yes	567	26.4	97.5	2.5
	No	1581	73.6		
Family diabetes	Yes	192	9.2	97.0	3
	No	1901	90.8		
Daily smoking	Yes	103	4.8	97.9	2.1
	No	2052	95.2		
Weekly using of fruit	Yes	2082	96.7	97.8	2.2
	No	72	3.3		
Place of life	Rural	870	40	100	0
	Urban	1288	60		
Marital status	Single	98	4.6	98.8	1.2
	Married	1974	92.6		
	Widow/widower	60	2.8		

## Discussion

The prevalence of IFG, diabetes and hyperglycaemia among participants in the current study were 7.8, 11.8 and 19.6 %, respectively and participants from urban area showed a higher prevalence of hyperglycaemia than those from the rural regions. A study from Iran in 2009 reported a prevalence of 16.3 % for diabetes in Yazd city and also reported that 14–23 % of the Iranian population, with the age higher than 30 years old, are suffering from diabetes or IFG [21]. These figures are higher than what have been indicated in the current study. More studies from Iran by Janghorbani et al., Azizi et al., and Larijani et al., during 1997–2005 [22–25], reported prevalence rates of 4.6–10 % for diabetes which are more closed to what has been reported by the current study compared to the study in Yazd. The distribution of diabetes in Iran has been the subject of several surveys. Disease prevalence rates, for all forms of diabetes, have been variously reported as 7–17 % in several adult urban populations. However, geographical prevalence is not uniform and the prevalence of type 2 diabetes mellitus has been reported at 3–5 % in rural communities. A screening program conducted at different locations of Iran revealed that nearly 50 % of people with type 2 diabetes were unaware of their conditions [24]. Another national cross-sectional survey among 70,981 Iranian citizens, aged 25–64 years, found that 7.7 % of adults aged 25–64 years (2 million adults) had diabetes, among whom one-half were undiagnosed. An additional 16.8 % (4.4 million) of Iranian adults have impaired fasting glucose [26]. Therefore, this high rate of lack of awareness for serum glucose status may increase the chances of diabetes complications among Iranian population.

The prevalence of type 2 diabetes appears to be high among Iranian people over 40 years old with a figure of 24 % which increases by 0.4 % each year after the age of 20 years. In a study, it was reported that the risk of type 2 diabetes was 1.7 % greater in women compared to men [27]. It seems that the crude prevalence is not an appropriate indicator, due to differences in age pyramids, and an age-adjusted or age-specific prevalence should be used for this purpose.

Though our results showed a higher diabetes prevalence rate in Ilam province compared to the overall rate of the country, this figure is still lower than the prevalence rates reported from some other provinces such as Yaz (14.01 %), Kerman (13.16 %), Qazvin (13.09 %), Bushehr (12.62 %), and Isfahan (12.19 %). On the other hand, this prevalence rate is much higher than that in Kurdistan (3.35 %), Lorestan (3.62 %), Gilan (4.45 %), Zanjan (4.62 %) and some other provinces [27]. These discrepancies may be associated with verity of diabetes –related risk factors in different geographical areas in Iran or differences in the methodology applied by different studies performed.

Studies from other parts of the world have reported either higher or lower prevalence of hyperglycaemia and IFG than the figure reported by the current study, which could be due to the effects of geographical conditions, nutritional habits, racial and genetically differences, customs, social behaviors and different life styles on diabetes and IFG. A study from the USA [4] in 2002, has reported a prevalence rate of 38 % for hyperglycaemia among hospitalized patients but another study investigated unrecognised diabetes among patients with coronary diseases and found that 46.6 % of patients had hyperglycaemia with no prior history of diabetes [5].

Amongst the top ten countries with the highest prevalence rates of diabetes, three are from the Middle East and North Africa Regions including Saudi Arabia, Kuwait, and Qatar with prevalence rates of 24, 23.1 and 22.9 %, respectively. Indeed, many of the countries in the Middle East and North Africa Regions have prevalence rates well above the global prevalence of 8.3 %, among which Egypt (15.6 %), Turkey (14.6 %), UAE (10 %) and Tunisia (9.2 %) have the highest prevalence rates, after the 3 above mentioned countries [28]. Iran with a total prevalence rate of 8.4 % has approximately a similar rate to the global figure. As diabetes is a disease with multifactorial causes, associated with social, behavioral, occupational, life style and other factors, the discrepancies in its prevalence rates among different geographical regions, nations, societies and even different races in a city could be justifiable.

Community based studies, similar to the current study, have reported different prevalence of hyperglycaemia. For example, Cowie and others from the USA have reported a prevalence rate of 26 % for IFG [9]; however, Meisinger and colleagues from Germany have reported an overall prevalence of 16 % for either diabetes or abnormalities of glucose metabolism [10] and Sakikawas’ study from Japan reported a figure of 12.9 % [11].

The above mentioned studies were some samples of the developed countries; however, studies from developing countries have reported similar discrepancies for hyperglycaemia prevalence. A study from south of India by Mohan in 2003, reported a prevalence rate of 5.9 % for IFG and 7.2 % for diabetes [14] but another study by Shera from Pakistan in 2010, reported a prevalence of total glucose intolerance of 16.7 % for males and 19.4 % for females [16].

The current study showed that the prevalence of hyperglycaemia and IFG among urban population was significantly higher compared to rural population. Though not significant, our study showed a higher prevalence of diabetes and IFG among males compared to females; however, other studies from Pakistan [16] and Colombia [12] reported a higher prevalence among females. A study by Sarah Wild and others in 2004 also reported a higher prevalence of diabetes among males compared to females [29].

According to the results of the current study, different cities of Ilam province have also shown different prevalence rates of diabetes and IFG from a minimum of 5.3 % in Aivan county to a maximum of 14.8 % in Mehran county for diabetes and between a minimum of 1.9 % in Aivan county to a maximum of 11.8 % in Chardavel county for IFG. These discrepancies may be related to the effects of geographical, customs, social behaviors and nutritional conditions on diabetes and IFG.

The prevalence of IFG, diabetes and hyperglycaemia among singles, married and widows were 6.1, 4.1, 10.2; 7.9, 12.3, 20.2 % and 10, 18.3, 28.3 %, respectively. This can indicate the effects of mean age on IFG and diabetes among married and widows compared to singles and the effect of age on IFG and diabetes is a recognized factor.

There was a significant difference between the prevalence of IFG, diabetes and hyperglycemia among those with hypertension compared to those without (10.5 %, 21.4 %, 31.9 % vs 7.5 %, 10.6 %, 18.1 % respectively). This difference and any relationship between the history of hypertension and aggravation of IFG or diabetes should be approved by future studies.

The current study showed a significantly higher prevalence of hyperglycaemia among those with a history of family diabetes (37 %) compared to those without (12.4 %). This difference has already been shown as a recognized factor by other references [29].

The prevalence of IFG, diabetes and hyperglycaemia was significantly higher among smokers, compared to non- smokers (11.7 %, 13.6 %, 25.3 % vs. 7.7 %, 11.7 %, 19.4 %, respectively). More studies need to approve the effects of this factor on diabetes and IFG.

There was a significantly positive relationship between age and diabetes or IFG. For example, the 9.8 and 24 % prevalence of diabetes among participants with the age group of 21–30 years and those with the age group of 71–80 years were reported respectively [29].

Another factor related to diabetes in the current study was height. The prevalence rate of diabetes was 5.6 % among the participants with a height of 150 cm or less and 18.1 % among those with a height above 170 cm. Considering a positive relationship between age and height, this process may be related to the effects of age on diabetes which has already been approved. Regardless of age, the pure effects of height on diabetes should be investigated by future studies.

There was a positive relationship between the prevalence of diabetes and waist size among men; for example, participants with a mean waist size of 70 cm had a diabetes prevalence of11.6 % compared to those with a mean waist above 102 cm who had a prevalence of 14.6 %. This could be due to accompany of fatness with higher waist size and the effects of fatness on diabetes has already been recognized [30].

This study was the first comprehensive population based survey for type 2 diabetes in Ilam province which could be used as a pilot study for any future interventional or preventive issues or academic researches in this province. A self-assessment predictive model for diabetes could be constructed upon the results of this study and we are now working on this model which could be applied by other provinces in the western part of Iran. This study also has some limitations such as lower proportion of recruited males and excess of participated women in the study, not a 100 % availability rate for all the variables, and not performing GTT for diabetes confirmation when FBS was higher than 126 mg/dl. Data collection and blood sampling were performed in the morning and in that particular time men were usually outside the home or not interested to participate in the study. However, there was a relatively large number of participants in this study and notwithstanding the excess number of women, there was still enough number of participated men for investigation and comparison of different variables.

## Conclusions

The regression analysis revealed that the most effective factors associated with IFG and diabetes were family history of diabetes, age, hypertension, marital status, place of life and smoking, respectively. The prevalence of IFG, diabetes and hyperglycemia among population living in Ilam province, west of Iran, were 7.8, 11.8 and 19.6 %, respectively which were increased with age.
